# Body Composition Variables as Radiographic Biomarkers of Clinical Outcomes in Metastatic Renal Cell Carcinoma Patients Receiving Immune Checkpoint Inhibitors

**DOI:** 10.3389/fonc.2021.707050

**Published:** 2021-07-09

**Authors:** Dylan J. Martini, T. Anders Olsen, Subir Goyal, Yuan Liu, Sean T. Evans, Benjamin Magod, Jacqueline T. Brown, Lauren Yantorni, Greta Anne Russler, Sarah Caulfield, Jamie M. Goldman, Bassel Nazha, Haydn T. Kissick, Wayne B. Harris, Omer Kucuk, Bradley C. Carthon, Viraj A. Master, Mehmet Asim Bilen

**Affiliations:** ^1^ Winship Cancer Institute of Emory University, Department of Hematology and Medical Oncology, Atlanta, GA, United States; ^2^ Massachusetts General Hospital, Department of Medicine, Boston, MA, United States; ^3^ Department of Hematology and Medical Oncology, Emory University School of Medicine, Atlanta, GA, United States; ^4^ Departments of Biostatistics and Bioinformatics, Emory University, Atlanta, GA, United States; ^5^ Northwestern University, Department of Medicine, Chicago, IL, United States; ^6^ Department of Pharmaceutical Services, Emory University School of Medicine, Atlanta, GA, United States; ^7^ Department of Urology, Emory University School of Medicine, Atlanta, GA, United States

**Keywords:** body composition, mRCC, immune checkpoint inhibitors, sarcopenia, adiposity, prognostic model, biomarkers

## Abstract

**Background:**

Immune checkpoint inhibitors (ICI) have revolutionized the treatment of metastatic renal cell carcinoma (mRCC). Biomarkers for mRCC patients treated with ICI are limited, and body composition is underutilized in mRCC. We investigated the association between body composition and clinical outcomes in ICI-treated mRCC patients.

**Methods:**

We performed a retrospective analysis of 79 ICI-treated mRCC patients at Winship Cancer Institute from 2015-2020. Baseline CT images were collected at mid-L3 and segmented using SliceOMatic v5.0 (TomoVision). Density of skeletal muscle (SM), subcutaneous fat, inter-muscular fat, and visceral fat were measured and converted to indices by dividing by height(m)^2^ (SMI, SFI, IFI, and VFI, respectively). Total fat index (TFI) was defined as the sum of SFI, IFI, and VFI. Patients were characterized as high *versus* low for each variable at gender-specific optimal cuts using overall survival (OS) as the primary outcome. A prognostic risk score was created based on the beta coefficient from the multivariable Cox model after best subset variable selection. Body composition risk score was calculated as IFI + 2*SM mean + SFI and patients were classified as poor (0-1), intermediate (2), or favorable risk (3-4). Kaplan-Meier method and Log-rank test were used to estimate OS and PFS and compare the risk groups. Concordance statistics (C-statistics) were used to measure the discriminatory magnitude of the model.

**Results:**

Most patients were male (73%) and most received ICI as first (35%) or second-line (51%) therapy. The body composition poor-risk patients had significantly shorter OS (HR: 6.37, p<0.001), PFS (HR: 4.19, p<0.001), and lower chance of CB (OR: 0.23, p=0.044) compared to favorable risk patients in multivariable analysis. Patients with low TFI had significantly shorter OS (HR: 2.72, p=0.002), PFS (HR: 1.91, p=0.025), and lower chance of CB (OR: 0.25, p=0.008) compared to high TFI patients in multivariable analysis. The C-statistics were higher for body composition risk groups and TFI (all C-statistics ≥ 0.598) compared to IMDC and BMI.

**Conclusions:**

Risk stratification using the body composition variables IFI, SM mean, SFI, and TFI may be prognostic and predictive of clinical outcomes in mRCC patients treated with ICI. Larger, prospective studies are warranted to validate this hypothesis-generating data.

## Background

Immune checkpoint inhibitors (ICI) have become an important option for the treatment of metastatic renal cell carcinoma (mRCC) over the past 5 years (1). Nivolumab, a programmed death protein-1 (PD-1) inhibitor, was the first ICI approved for mRCC in 2015 (2). Since that time, several ICI-based combination treatment regimens have been approved for treatment-naïve mRCC including nivolumab plus ipilimumab, pembrolizumab plus axitinib, avelumab plus axitinib, and nivolumab plus cabozantinib (2). Despite the increased use of ICI for mRCC, a subset of patients do not respond to treatment with ICI-based treatment regimens. Furthermore, biomarkers to help determine which patients are more likely to respond to treatment with ICIs are limited. Hence, the identification of robust clinical biomarkers of response to ICI in mRCC is an unmet need in the field of genitourinary oncology.

At this time, body composition is under-studied as a biomarker in mRCC patients. The investigation of markers of body composition as prognostic biomarkers in mRCC patients has primarily been focused on body mass index (BMI) (3). Additionally, increased BMI has been shown to be a favorable prognostic factor in patients with several malignancies treated with ICIs including non-small cell lung cancer (NSCLC), melanoma, and mRCC (4–6). There is a growing body of literature investigating sarcopenia, measured by skeletal muscle index (SMI), as a possible biomarker. Although the majority of these studies have been performed in the peri-operative setting for mRCC patients undergoing nephrectomy (7, 8), sarcopenia has also been shown to be a significant predictor of OS in mRCC (9). Additionally, subcutaneous fat index (SFI) was found to be an independent predictor of mortality in mRCC patients treated with sunitinib (10). Other markers of adiposity such as total fat index (TFI), visceral fat index (VFI), or inter-muscular fat index (IFI) have not been found to be associated with clinical outcomes in mRCC patients. Furthermore, there have been no studies investigating markers of adiposity in mRCC patients treated with ICIs.

In this study, we performed a comprehensive investigation of the association between radiographic markers of body composition and clinical outcomes in mRCC patients treated with ICI-based treatment regimens. We used markers of both sarcopenia and adiposity to create a novel risk scoring system in this cohort of patients. We also compared the predictive value of our model to the validated international mRCC database consortium (IMDC) criteria and BMI. Importantly, we also assessed the association between a composite marker of adiposity, total fat index (TFI), in this cohort of ICI-treated mRCC patients. We hypothesize that the findings from this study may provide evidence for body composition markers to be considered for inclusion in updated prognostic risk models for mRCC patients treated with ICIs. These results may be helpful for practicing oncologists in the academic or community setting given the increasing use of ICIs for several malignancies including mRCC.

## Methods

### Patients and Data

We performed a retrospective analysis of 79 ICI-treated mRCC patients at Winship Cancer Institute of Emory University from 2015-2020. This study was approved by the Emory University Institutional Review Board. Inclusion criteria for this study were: (1) confirmed histologic diagnosis of RCC, (2) receipt of at least 1 dose of ICI and (3) availability of computed tomography (CT) scans within 2 months of ICI-initiation. Baseline CT images were collected at mid-L3 and segmented using SliceOMatic v5.0 (TomoVision) by one author (DJM). Adequate training was confirmed by an intra-observer variation < 1.3%. We collected the density of skeletal muscle (SM), subcutaneous fat, inter-muscular fat, and visceral fat using the following Hounsfield Unit (HU) references ranges (-29 to + 150 HU for skeletal muscle, -190 HU to -30 for subcutaneous and inter-muscular fat, -150 to -50 HU for visceral fat) (11, 12). Each density was converted to an index by dividing by height (m)^2^ (SMI, SFI, IFI, and VFI, respectively). Total fat index (TFI) was defined as the sum of SFI, IFI, and VFI. Additional clinical data was collected including demographics, Eastern Cooperative Oncology Group Performance Status (ECOG PS), number and type of prior systemic therapies, sites of metastatic disease, and baseline BMI. IMDC criteria were used to characterize patients as favorable, intermediate, or poor-risk (13).

We used three different measures of clinical outcomes: overall survival (OS), progression-free survival (PFS), and clinical benefit (CB). OS and PFS were calculated as the number of months elapsed from the first dose of ICI to date of death or radiographic or clinical progression, respectively. CB was defined as a best radiographic response of complete response (CR), partial response (PR), or stable disease (SD) for ≥ 6 months per response evaluation criteria in solid tumors version 1.1 (RECISTv1.1) (14). CB is a two-level variable, in which the responder is defined as having a best radiographic response of CR, PR, or SD ≥ 6 months and the non-responders were defined as PD or non-evaluable.

### Statistical Analysis

Statistical analysis was performed using SAS Version 9.4, and SAS macros, which was developed by the Biostatistics Shared Resource at Winship Cancer Institute (15). The significance level was set at p < 0.05 and descriptive statistics for each variable were reported. The univariate association (UVA) of each covariate with OS and PFS was tested by proportional hazard model with a reported hazard ratio (HR) and its 95% confidence interval (CI) being reported. In the analysis for CB, we used logistic regression and modeled the probability of response to find the odds ratio (OR). Each fat index was characterized as high *versus* low for each variable at gender-specific optimal cuts using overall survival (OS) as the primary outcome through a bias-adjusted log-rank test searching algorithm (16). A prognostic risk score was created based on the beta coefficient from the multivariable Cox model (MVA) after best subset variable selection (17). Body composition risk score was calculated as IFI + 2*SM mean + SFI, and patients were classified as poor (0-1), intermediate (2), or favorable risk (3-4). The prediction performance by BMI, IMDC Risk Group, and our body composition risk score was measured and compared by Uno’s concordance statistics (C-statistics) (18). The area under the curve (AUC) was reported for the discrimination for CB analysis. Kaplan-Meier method and Log-rank test were used to estimate OS and PFS and compare the risk groups.

## Results

### Demographic Information and Baseline Disease Characteristics

Descriptive statistics for demographic information and baseline disease characteristics are presented in **Table 1**. Most patients were males (73.4%) and median age was 61.0 years old. The majority of patients were Caucasian (n=61, 77.2%) and more than one-fifth (n=17, 21.5%) were African Americans. Most patients had clear cell RCC (n=55, 74.3%) and patients were primarily intermediate (54%) or poor-risk (30%) per IMDC criteria. More than one-quarter of patients (n=19, 27) had three or more metastatic sites at baseline. The majority of patients received no prior systemic therapy (35%) or one (51%) prior line of systemic therapy prior to initiating ICI. Anti-PD-1 monotherapy was the most common treatment regimen (n=47, 59.5%), while 40.5% received an ICI combination regimen.

**Table 1:** Demographics and baseline characteristics.

Variable		n (%)
Gender	Male	58 (73.4)
	Female	21 (26.6)
Race	White	61 (77.2)
	Black	17 (21.5)
	Asian	1 (1.3)
ECOG PS	0-1	58 (75.3)
	2+	19 (24.7)
	Missing	2
ccRCC	Yes	55 (74.3)
	No	19 (25.7)
	Missing	5
Anti-PD-1 Monotherapy	Yes	47 (59.5)
	No	32 (40.5)
Prior Lines of Therapy	0	28 (35.4)
	1	40 (50.6)
	2	7 (8.9)
	3+	4 (5.1)
Number of distant metastatic sites	1	12 (15.2)
	2	27 (34.2)
	3+	40 (50.6)
IMDC Risk Groups	Favorable	12 (15.2)
	Intermediate	43 (54.4)
	Poor	24 (30.4)
Baseline BMI (Median: 26.2)	≤25	29 (37.2)
	>25	49 (62.8)
	Missing	1
Median (Optimal Cut-Off) Muscle and Adipose Variables	SMI	M: 44.0, F: 39.2
	Attenuated SM Mean	M: 35.1, F: 34.4
	SFI	M: 51.4, F: 69.8
	IFI	M: 4.4, F: 7.8
	VFI	M: 35.2, F: 37.4
	TFI	M: 98.7, F: 94.3
Median Age: 61.0 years		

ECOG PS, Eastern cooperative oncology group performance status; ccRCC, clear cell renal cell carcinoma; BMI, body mass index; SMI, skeletal muscle index; SM, skeletal muscle; SFI, subcutaneous fat index; IFI, inter-muscular fat index; VFI, visceral fat index.

### Risk Group Analysis

**Figure 1:**
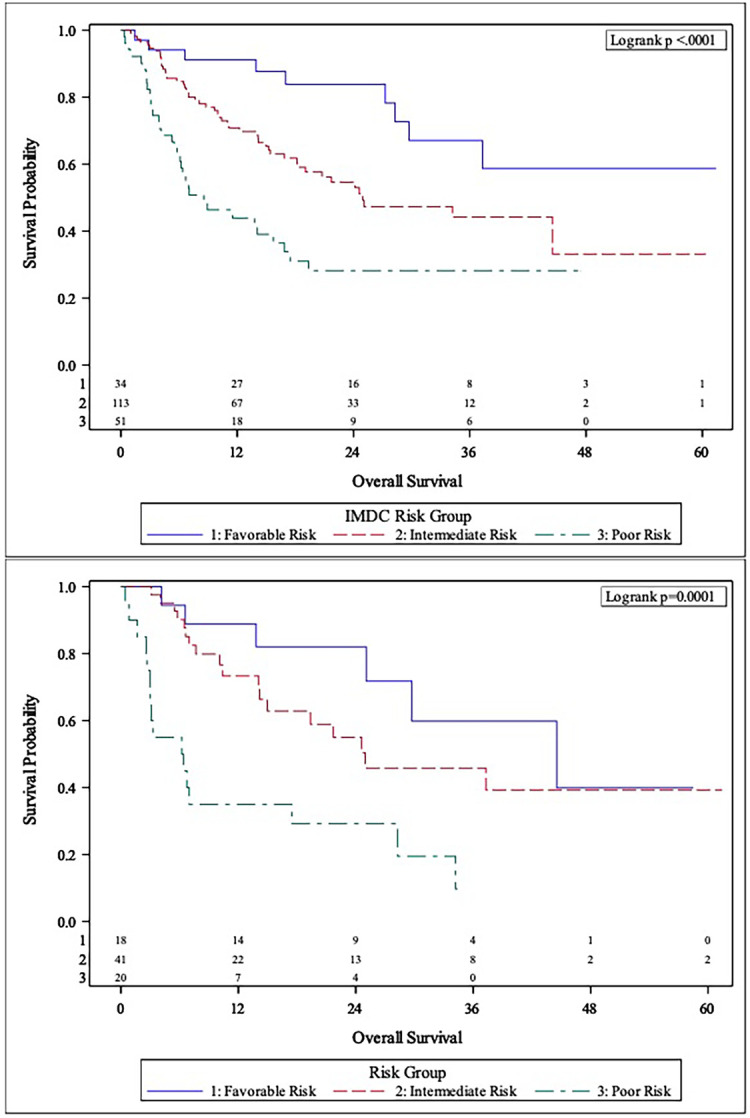
Comparison of Kaplan-Meier curves between IMDC risk groups (Top panel) and body composition risk groups (Bottom panel) for overall survival (OS).

**Figure 2:**
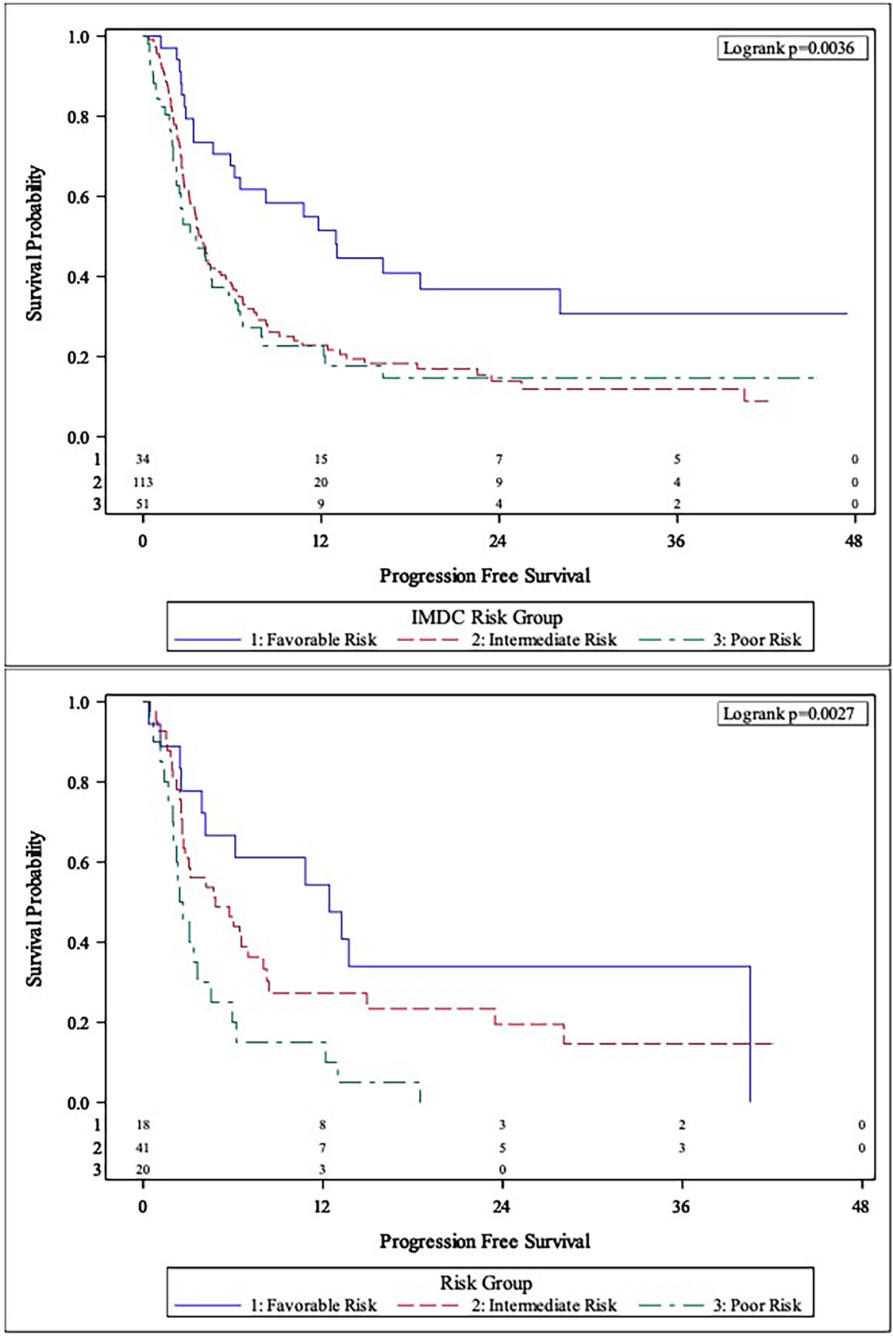
Comparison of Kaplan-Meier curves between IMDC risk groups (Top Panel) and body composition risk groups (Bottom Panel) for progression-free survival (PFS).

The MVA of the association between body composition and TFI with clinical outcomes is presented in **Table 2**. The body composition poor-risk patients had significantly shorter OS (HR: 6.37, CI: 2.40-16.92, p<0.001), PFS (HR: 4.19, CI: 1.87-9.42, p<0.001), and lower chance of CB (OR: 0.23, CI: 0.05-0.96, p=0.044) compared to favorable risk patients in MVA. Intermediate risk patients also showed a trend towards shorter PFS (HR: 2.05, CI: 0.98-4.29, p=0.057) compared to favorable risk patients. There was a step-wise decline in median OS and PFS from favorable risk to intermediate risk to poor risk patients per Kaplan Meier estimation (OS: 44.5 months *versus* 24.6 months *versus* 6.3 months; PFS: 12.4 months *vs.* 4.8 months *vs.* 2.5 months, **Table 2** and **Figures 1**, **2** **).** The C-statistics were higher for our body composition risk groups compared to IMDC and BMI for all three clinical outcomes (**Table 3** and **Supplemental Figure 1**).

**Table 2:** MVA* of association between body composition risk groups and TFI with clinical outcomes.

	OS		PFS		CB	
	HR (CI)	p-value	HR (CI)	p-value	OR (CI)	p-value
**Body Composition Risk Group Analysis**						
**Poor Risk:** Risk Score = 0-1 *n = 20*	6.37 (2.40-16.92)	<0.001**	4.19 (1.87-9.42)	<0.001**	0.23 (0.05-0.96)	0.044**
	**Median Survival:** 6.3 months		**Median Survival:** 2.5 months			
	**24-Month Survival:** 29.2%		**12-Month Survival:** 15.0%			
**Intermediate Risk:** Risk Score = 2	1.56 (0.61-3.95)	0.350	2.05 (0.98-4.29)	0.057	0.49 (0.15-1.59)	0.238
*n *= 42						
	**Median Survival:** 24.6 months		**Median Survival:** 4.8 months			
	**24-Month Survival:** 53.1%		**12-Month Survival:** 26.6%			
**Favorable Risk:** Risk Score = 3-4	1		1		1	
*n *= 18						
	**Median Survival:** 44.5 months		**Median Survival:** 12.4 months			
	**24-Month Survival:** 82.1%		**12-Month Survival:** 54.3%			
Categorical Total Fat Index (TFI) Analysis***						
**Low**n = 34	2.72 (1.43-5.17)	0.002**	1.91 (1.09-3.35)	0.025**	0.25 (0.09-0.70)	0.008**
**High**	1		1		1	
n = 45						

* MVA controlled for race, gender, clear cell RCC, Baseline BMI, Age, anti-PD-1 monotherapy, IMDC risk groups and number of prior lines of therapy.

**Statistically significant at the level of p < 0.05.

***High vs low TFI determined by optimal cut analysis.

Bold p-values represent statistically significant values.

**Table 3:** Comparison of C-statistics between body composition risk groups, TFI, IMDC, and BMI.

	OS C-Statistic	p-value Comparison to IMDC	p-value Comparison to BMI	PFS C-Statistic	p-value (comparison to IMDC)	p-value (comparison to BMI)	CB C-statistic	p-value (comparison to IMDC)	p-value (comparison to BMI)
**Risk Group**	0.648	0.749	0.228	0.612	0.738	0.313	0.637	0.513	0.136
**TFI**	0.626	0.987	0.186	0.598	0.878	0.174	0.646	0.400	0.012*
**IMDC**	0.613	Not Available		0.575	Not Available		0.584	Not Available	
**BMI**	0.562			0.544			0.522		

*Statistically significant at the level of p < 0.05.

Bold p-values represent statistically significant values.

### Total Fat Index (TFI) Analysis

The categorical TFI analysis investigating the association with clinical outcomes is also presented in **Table 2**. Patients with low TFI had significantly shorter OS (HR: 2.72, CI: 1.43-5.17, p=0.002), PFS (HR: 1.91, CI: 1.09-3.35, p=0.025), and lower chance of CB (OR: 0.25, CI: 0.09-0.70, p=0.008) compared to high TFI patients in MVA. High TFI patients had significantly longer median OS (44.5 *vs.* 14.1 months, p=0.0012) and PFS (8.4 *vs.* 2.9 months, p=0.0015) compared to low TFI patients per Kaplan-Meier estimation (**Supplemental Figure 2**). Additionally, TFI had higher C-statistics for predicting OS, PFS, and CB compared to both IMDC and BMI (**Table 3**). Notably, TFI was significantly better at predicting clinical benefit compared to BMI (C-statistics: 0.646 *vs.* 0.522, p=0.012).

## Discussion

Overall, we found that increased adiposity and increased attenuated SM mean were associated with improved outcomes in this cohort of mRCC patients treated with ICI-based treatment regimens. We used two different methods to present the association of adiposity with clinical outcomes. First, we created a novel prognostic risk scoring system which included two measures of adiposity: SFI and VFI. In a secondary analysis, we showed that low TFI was significantly associated with worse outcomes. This is an important study in that it provides hypothesis-generating data regarding the prognostic risk associated with certain radiographic measurements of body composition. Although BMI has been associated with improved outcomes in mRCC patients, this is the first and most comprehensive study investigating risk associated with different components of adipose tissue and skeletal muscle on clinical outcomes in ICI-treated mRCC patients. Importantly, this study highlights the prognostic value of CT imaging in measuring adiposity in mRCC patients.

The hypothesis-generating data presented in this study has important clinical implications for mRCC patients initiating therapy with ICI-based treatment regimens. Namely, the significant association of body composition variables including SFI, IFI, attenuated SM mean, and TFI highlight the under-utilization of data collected by CT imaging in oncology patients treated with immunotherapy. These results add to a growing body of literature supporting the inclusion of body composition variables in updated prognostic and predictive models in mRCC patients treated with ICI-based treatment regimens. Radiographic body composition measures are particularly attractive as clinical biomarkers because baseline imaging is performed as standard of care for patients starting on a new line of systemic therapy. A recent study by Higgins et al. found that there was high correlation for adipose tissue and muscle measures between CT images and magnetic resonance imaging (MRI) (19). It should be noted, that there was a bias towards 10.34% lower measures of subcutaneous fat density on MRI which contributed to our decision to only including patients with baseline CT imaging in this study. Future studies are required to standardize the process of segmenting MRI images before the results from this study may be generalized to patients undergoing baseline MRI prior to ICI-initiation.

Increased subcutaneous fat was associated with improved outcomes in this study, which is consistent with our group’s previous findings of an association between increased SFI and longer OS and PFS in phase 1 clinical trial patients treated with immunotherapy (20). This is the first study, to our knowledge, to find an association between SFI and ICI-treated mRCC patients. This finding highlights the obesity paradox in cancer, in which obesity has been shown to contribute to carcinogenesis, yet obese patients may be more likely to have improved outcomes to treatment (21). Increased BMI has been associated with improved outcomes in ICI-treated NSCLC and melanoma patients (4, 5). This association was also described in patients with mRCC (6). A large retrospective study (n=736) found similar tumor mutational burden and genomic alterations between high and low BMI mRCC patients treated with ICIs (6). One possible explanation for this association is the fact that adipocyte PD-L1 expression increases during adipogenesis, suggesting that adiposity promotes tumor immune evasion which may be reversed by ICI-based treatment regimens *via* increased effector T-cells (22, 23). Surprisingly, we also found that increased IFI was associated with improved outcomes and was one of the variables chosen for inclusion in our risk group analysis. It is possible that increased IFI in this population is a reflection of total body adiposity, given that the Pearson correlation coefficients with SFI and TFI were 0.637 and 0.655, respectively (both p<0.001, **Supplemental Figure 3** **)**. Taken together, we provide evidence that radiographic markers of adiposity such as SFI and IFI may be clinical biomarkers of improved outcomes in ICI-treated mRCC patients.

**Figure 3:**
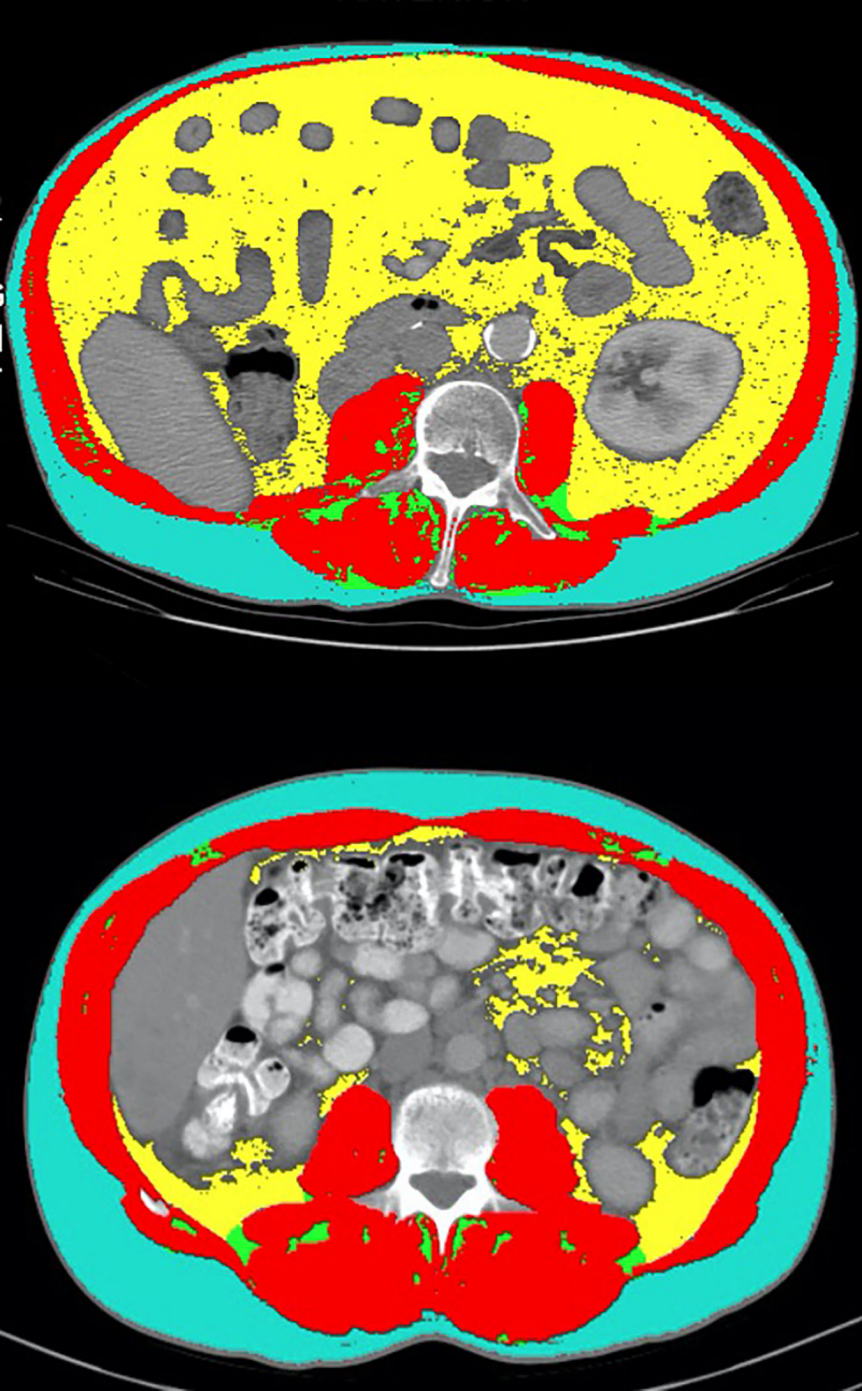
Comparison of select CT segmentation results between two patients with similar BMI but disparate TFI and clinical outcomes. The first patient (top panel) had a BMI of 24.9 and a TFI of 119.97 (high). He had a best radiographic response of partial response on treatment with anti-PD-1 combination therapy. The second patient (bottom panel) had a BMI of 24.4, but a TFI of 44.88 (low). This patient had a best radiographic response of progressive disease on treatment with anti-PD-1 combination therapy.

An important finding with significant clinical relevance presented in this study is that TFI was independently associated with OS, PFS, and CB in MVA. Additionally, the C-statistics for predicting clinical outcomes were all higher for TFI than BMI, including a significantly higher C-statistics for predicting CB. This highlights the possible predictive and prognostic value of TFI which has not been previously described in the literature for mRCC patients. One possible explanation for this observation in this cohort is that adipose tissue is a secondary lymphoid organ and houses populations of T-cells (24). The value of TFI as a potential biomarker is highlighted in **Figure 3**, which compared two patients with similar BMI but disparate TFI and clinical outcomes on treatment with anti-PD-1 combination therapy. The first patient had a BMI of 24.9 and a high TFI of 119.97. He had a best radiographic response of PR on treatment. The second patient had a BMI of 24.4, but a low TFI of 44.88. This patient had a best radiographic response of progressive disease on anti-PD-1 combination treatment. This radiographic representation combined with the higher C-statistics of TFI compared to BMI suggest that TFI may be a better marker of total body adiposity than BMI in mRCC patients treated with ICI.

**Figure 4:**
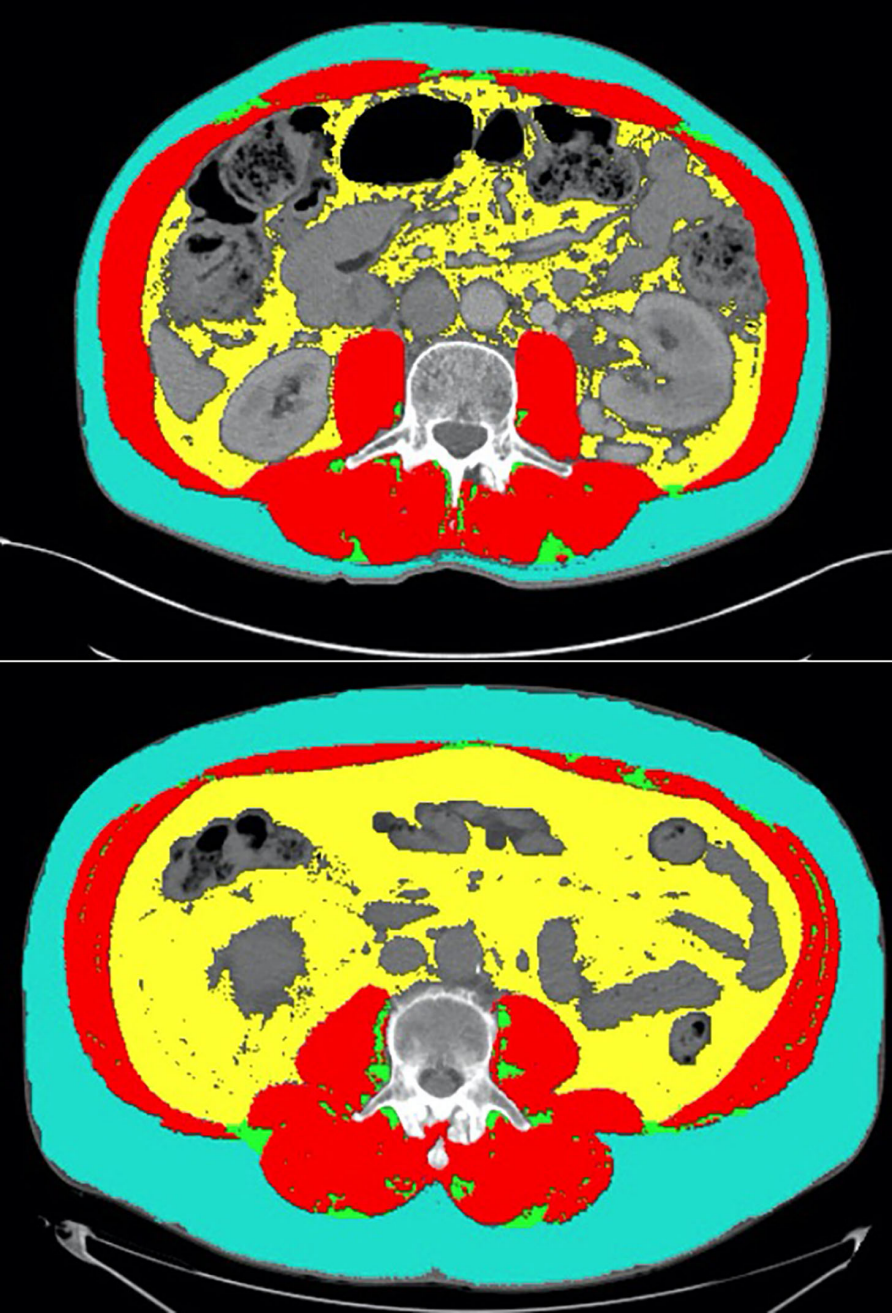
Representative CT segmentation results from two patients with similar SMI but disparate attenuated SM mean. The first patient (top panel) had an SMI of 52.48 and an attenuated SM mean of 41.45 (high). He had a partial response on treatment with anti-PD-1 monotherapy and had remained progression free for over 9 months at the time of last follow-up. The second patient (bottom panel) had a SMI of 59.38 and an attenuated SM mean of 24.97 (low). This patient had progressive disease as his best radiographic response to anti-PD-1 monotherapy.

Interestingly, we found that attenuated SM mean was a better prognostic marker than SMI in our analysis. Decreased attenuated SM mean has been used as a marker of myosteatosis, given that it is inversely associated with intramuscular lipid deposition (25). Hence, we found that increased myosteatosis was associated with poor outcomes. This adds to data from a recent study which found that high skeletal muscle gauge (SMI x attenuated SM mean) was modestly associated with improved outcomes in melanoma patients treated with ICI combination therapy with nivolumab and ipilimumab (26). This is also consistent with our findings in a separate analysis that increased attenuated SM mean was significantly associated with improved outcomes in ICI-treated urothelial carcinoma patients (27). This provides further support for attenuated SM mean as an adjunctive biomarker to SMI in quantifying risk associated with sarcopenia and myosteatosis in ICI-treated patients with malignancies of the GU tract. The clinical utility of myosteatosis as a prognostic marker is highlighted by comparing two patients treated with anti-PD-1 monotherapy who had similar baseline SMI, but disparate attenuated SM mean and clinical outcomes (**Figure 4** **)**. One of the patients (top panel) had an SMI of 52.48, but had an attenuated SM mean of 41.45, which is above the optimal cut in our analysis. He had a PR on treatment with anti-PD-1 monotherapy and had remained progression-free for over 9 months at the time of last follow-up. The second patient had a SMI of 59.38 and an attenuated SM mean of 24.97, which is below the optimal cut. This patient had progressive disease as his best radiographic response to anti-PD-1 monotherapy. This is an example of how attenuated SM mean can be used as an adjunct biomarker in analyzing the quality of skeletal muscle on CT imaging.

There are limitations to this study that should be noted. First, this is a heterogenous population of mRCC patients which included all patients who received at least one dose of ICI regardless of RCC subtype or line of therapy that ICI was received. We attempted to diminish the effect of these variables in our analyses by controlling for several baseline disease characteristics in MVA. Additionally, this is a relatively small sample size and the results presented in this study should be validated in a larger study. This is also a retrospective analysis which is vulnerable to selection bias, although we included all patients with available CT imaging and receipt of at least 1 dose of ICI with adequate clinical data availability. Future studies may explore the relationship between radiographic measures of body composition and metabolomic data in mRCC patients treated with ICI. Additionally, investigation of the predictive and prognostic value of our body composition risk score in patients treated with targeted therapy may provide insight into whether our system is specific for ICI-treated patients.

## Conclusions

Risk stratification using the body composition variables IFI, SM mean, SFI, and TFI may be prognostic and predictive of clinical outcomes in mRCC patients treated with ICI. These variables may be considered in updated prognostic models for ICI-treated mRCC patients. Larger, prospective studies are warranted to validate this hypothesis-generating data.

## Data Availability Statement

The original contributions presented in the study are included in the article/**Supplementary Material**. Further inquiries can be directed to the corresponding author.

## Ethics Statement

The studies involving human participants were reviewed and approved by Emory University IRB. Written informed consent for participation was not required for this study in accordance with the national legislation and the institutional requirements.

## Author Contributions

DM, SG, YL, and MB were involved in the study design and concept, statistical analysis, and drafting the manuscript. DM, SC, and MB were involved in the identification and selection of patients. DM collected and analyzed all CT images. DM, AO, SE, and BM were involved in data collection and quality control. All other authors were involved in the care of the patients included in this study. All authors were involved in the review and editing of the manuscript. All authors contributed to the article and approved the submitted version.

## Funding

Research reported in this publication was supported in part by the Breen Family Foundation and The Biostatistics and Bioinformatics Shared Resource of Winship Cancer Institute of Emory University and NIH/NCI under award number P30CA138292. The content is solely the responsibility of the authors and does not necessarily represent the official views of the National Institutes of Health.

## Conflict of Interest

BN has served as a member of the advisory board for Exelixis. BC has a consulting/advisory role with Astellas Medivation, Pfizer, and Blue Earth Diagnostics and receives travel accommodations from Bristol-Myers Squibb. MB has acted as a paid consultant for and/or as a member of the advisory boards of Exelixis, Bayer, BMS, Eisai, Pfizer, AstraZeneca, Janssen, Calithera Biosciences, Genomic Health, Nektar, and Sanofi and has received grants to his institution from Xencor, Bayer, Bristol-Myers Squibb, Genentech/Roche, Seattle Genetics, Incyte, Nektar, AstraZeneca, Tricon Pharmaceuticals, Genome & Company, AAA, Peloton Therapeutics, and Pfizer for work performed as outside of the current study.

The remaining authors declare that the research was conducted in the absence of any commercial or financial relationships that could be construed as a potential conflict of interest.

## Supplementary Material

Supplementary Table 1 Univariable analysis of muscle and adipose variables and clinical outcomes. *Patients dichotomized as high *vs.* low at optimal cut. **Statistically significant at p < 0.05. SMI, skeletal muscle index; IFI, inter-muscular fat index; SM, skeletal muscle; SFI, subcutaneous fat index; VFI, visceral fat index; BMI, body mass index.

Supplementary Figure 1 Receiver operating characteristic (ROC) curve comparison between body composition risk groups (Top panel) and TFI (Bottom panel) with IMDC risk group and BMI.

Supplementary Figure 2 Kaplan-meier curves for high *vs.* low total fat index (TFI) for overall survival (OS, top panel) and progression-free survival (PFS, bottom panel).

Supplementary Figure 3 Scatter plots of intermuscular fat (IFI) *versus* subcutaneous fat index (SFI, top panel) and total fat index (TFI, bottom panel).
