# Supplementary material for: Body Composition Variables as Radiographic Biomarkers of Clinical Outcomes in Metastatic Renal Cell Carcinoma Patients Receiving Immune Checkpoint Inhibitors
Source: Front Oncol. 2021 Jul 9;11:707050. doi: 10.3389/fonc.2021.707050 (PMC8299332; doi:10.3389/fonc.2021.707050)
Supplement: Supplementary Table 1 — Univariable analysis of muscle and adipose variables and clinical outcomes. *Patients dichotomized as high vs. low at optimal cut. **Statistically significant at p < 0.05. SMI, skeletal muscle index; IFI, inter-muscular fat index; SM, skeletal muscle; SFI, subcutaneous fat index; VFI, visceral fat index; BMI, body mass index. [file Table_1.docx]

**Supplemental Table 1:** Univariate analysis of muscle and adipose variables and clinical outcomes

| **Variable** | **Category*** | **OS** | | **PFS** | | **CB** | |
| --- | --- | --- | --- | --- | --- | --- | --- |
|  |  | **HR (CI)** | **p-value** | **HR (CI)** | **p-value** | **OR (CI)** | **p-value** |
| **SMI** | Low  n=27 | 1.59  (0.85-2.99) | 0.149 | 1.27  (0.76-2.12) | 0.360 | 0.79  (0.30-2.08) | 0.627 |
|  | High  n=52 | 1 |  | 1 |  | 1 |  |
| **IFI** | Low  n=43 | 1.57  (0.83-2.97) | 0.163 | 1.46  (0.88-2.41) | 0.142 | 0.55  (0.22-1.38) | 0.201 |
|  | High  n=36 | 1 |  | 1 |  | 1 |  |
| **TFI** | Low  n=34 | 2.73  (1.45-5.14) | 0.002** | 2.25  (1.35-3.77) | 0.002** | 0.27  (0.10-0.74) | 0.011** |
|  | High  n=45 | 1 |  | 1 |  | 1 |  |
| **Attenuated SM Mean** | Low  n=46 | 1.89  (0.97-3.66) | 0.061 | 1.38  (0.83-2.30) | 0.210 | 0.87  (0.34-2.21) | 0.769 |
|  | High  n=33 | 1 |  | 1 |  | 1 |  |
| **SFI** | Low  n=33 | 2.06  (1.10-3.85) | 0.024** | 2.02 (1.21-3.37) | 0.007** | 0.24 (0.09-0.66) | 0.006** |
|  | High  n=46 | 1 |  | 1 |  | 1 |  |
| **VFI** | Low  n=35 | 1.50  (0.81-2.80) | 0.199 | 1.94  (1.18-3.21) | 0.010** | 0.41  (0.16-1.07) | 0.070 |
|  | High  n=44 | 1 |  | 1 |  | 1 |  |
| **BMI** | ≤ 25  n=29 | 1.75  (0.93-3.30) | 0.082 | 1.38  (0.82-2.32) | 0.219 | 0.82  (0.31-2.17) | 0.690 |
|  | >25  n=49 | 1 |  | 1 |  | 1 |  |

*Patients dichotomized as high vs. low at optimal cut

**Statistically significant at p<0.05

SMI: skeletal muscle index, IFI: inter-muscular fat index, SM: skeletal muscle, SFI: subcutaneous fat index, VFI: visceral fat index, BMI: body mass index
