# Supplementary figures and images for: Body Composition Variables as Radiographic Biomarkers of Clinical Outcomes in Metastatic Renal Cell Carcinoma Patients Receiving Immune Checkpoint Inhibitors
Source: Front Oncol. 2021 Jul 9;11:707050. doi: 10.3389/fonc.2021.707050 (PMC8299332; doi:10.3389/fonc.2021.707050)

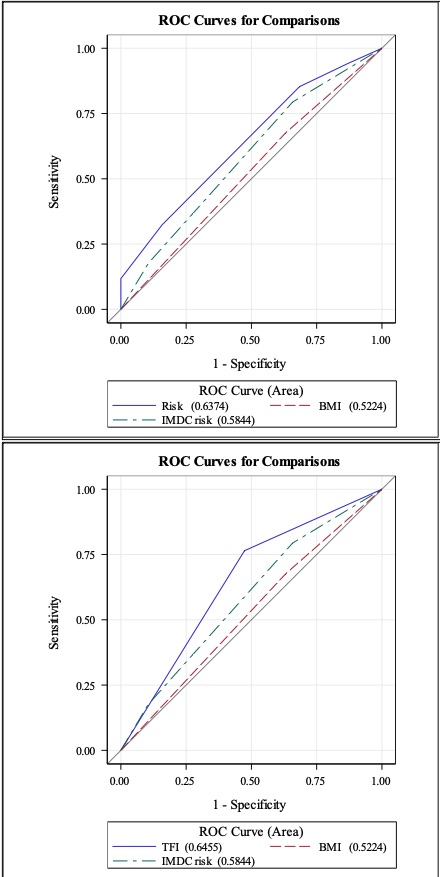

Supplement: Supplementary Figure 1 — Receiver operating characteristic (ROC) curve comparison between body composition risk groups (Top panel) and TFI (Bottom panel) with IMDC risk group and BMI. [file Image_1.jpeg]

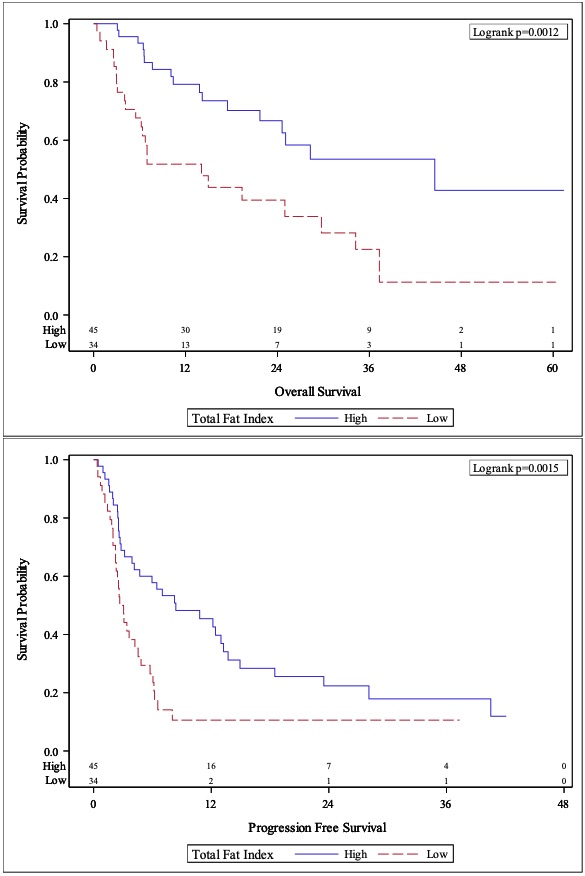

Supplement: Supplementary Figure 2 — Kaplan-meier curves for high vs. low total fat index (TFI) for overall survival (OS, top panel) and progression-free survival (PFS, bottom panel). [file Image_2.jpeg]

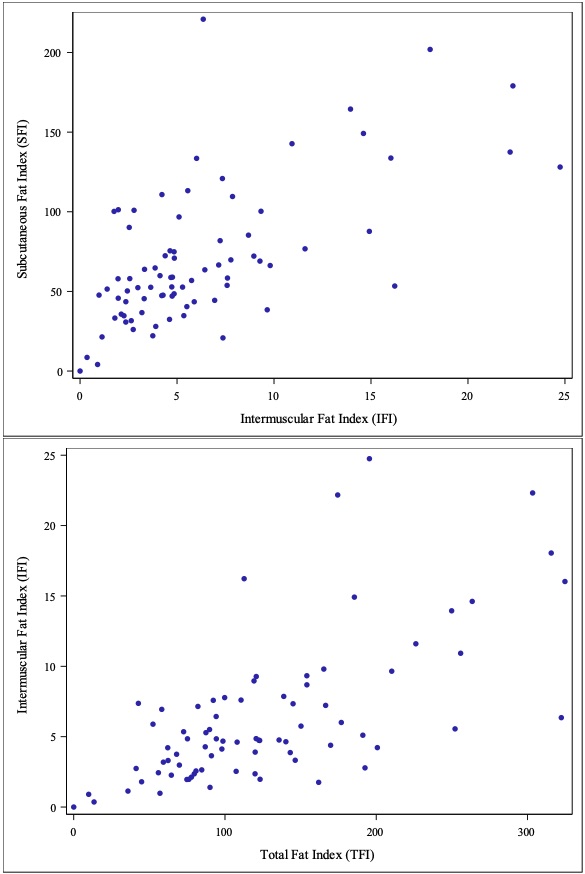

Supplement: Supplementary Figure 3 — Scatter plots of intermuscular fat (IFI) versus subcutaneous fat index (SFI, top panel) and total fat index (TFI, bottom panel). [file Image_3.jpeg]
